# Bi-Functional Biobased Packing of the Cassava Starch, Glycerol, Licuri Nanocellulose and Red Propolis

**DOI:** 10.1371/journal.pone.0112554

**Published:** 2014-11-10

**Authors:** Samantha Serra Costa, Janice Izabel Druzian, Bruna Aparecida Souza Machado, Carolina Oliveira de Souza, Alaíse Gil Guimarães

**Affiliations:** 1 Faculty of Pharmacy, Federal University of Bahia, Rua Barão de Geremoabo, s/n, Ondina, CEP 40171-970, Salvador, Bahia, Brasil; 2 Faculty of Pharmacy, Department of Bromatological Analysis, Federal University of Bahia, Rua Barão de Geremoabo, s/n, Ondina, CEP 40171-970, Salvador, Bahia, Brasil; 3 Faculty of Technology, SENAI/CIMATEC, Serviço Nacional de Aprendizagem Industrial - SENAI, Av. Orlando Gomes, n° 1845, Piatã, CEP 41650-010, Salvador, Bahia, Brasil; Tsinghua University, China

## Abstract

The aim of this study was to characterize and determine the bi-functional efficacy of active packaging films produced with starch (4%) and glycerol (1.0%), reinforced with cellulose nanocrystals (0–1%) and activated with alcoholic extracts of red propolis (0.4 to 1.0%). The cellulose nanocrystals used in this study were extracted from licuri leaves. The films were characterized using moisture, water-activity analyses and water vapor-permeability tests and were tested regarding their total phenolic compounds and mechanical properties. The antimicrobial and antioxidant efficacy of the films were evaluated by monitoring the use of the active films for packaging cheese curds and butter, respectively. The cellulose nanocrystals increased the mechanical strength of the films and reduced the water permeability and water activity. The active film had an antimicrobial effect on coagulase-positive staphylococci in cheese curds and reduced the oxidation of butter during storage.

## Introduction

The food industry seeks to develop products with quality and safety to meet a consumer market increasingly demanding. Thus, besides the application of good hygiene and sanitation practices, the packaging of the product in adequate to protect and conserve food during periods of storage and marketing packaging is required, assuring the consumer to purchase a healthy product [1].

Among the major types of containers for food and are flexible plastic films, which have excellent mechanical and gas barrier properties and water vapour [2], [3].

It has been estimated that 2% of all plastics eventually reach the environment, thus contributing considerably to a currently acute ecological problem. [4] In this context, packaging is seen as a major contributor to the environmental impact, since over a third of current plastics production is used to make them, and too because its relativity short life cycle. [4], [5] In addition, recent increases in the cost of raw petroleum have led to a dramatic increase in the cost of plastics. Hence, plastic pollution have driven the development of biobased packing with biodegradable polymers derived from renewable resources, that are kinds of environmentally-friendly materials, which can be degraded into carbon dioxide and water by microorganisms in natural environment [2], [3], [4].

Lately, numerous studies [3], [4], [6], [7] have been undertaken to provide alternative packaging made from renewable agricultural materials, such as starch. The starch based films are transparent, non-toxic, have moderately low permeability to oxygen and moisture barrier. [6], [7] However, the low mechanical resistance and high sensitivity to water restricts the use of these films, especially in foods with high moisture.

In this context, research has been conducted in an attempt to incorporate into the biodegradable matrices, organic nanoparticles such as cellulose nanocrystals (CNCs) or nanocellulose, which can improve the mechanical properties of the films. [2], [7] These nanoparticles are obtained from different sources of natural fibers such as cotton, wood, sisal, bamboo, coconut and sugarcane bagasse, sugarcane, among others [7].

Besides the possibility of becoming active biodegradable films with nanoparticles, there is a tendency to activate them bioactive compounds, resulting in active films reinforced mechanically retaining the biodegradability. [4], [7] The antimicrobial films, and antioxidants, for example, can control the development of specific species of microorganisms and enhance the oxidative stability of the packaged product. [8], [9], [10], [11], [12].

Most active packaging marketed have synthetic additives, as butilhidroxitolueno (BHT) and polihiroxialcanoatos (BHA), which despite their proven effectiveness, have restrictions on their use, as these components can migrate into food, posing a potential health risk. Thus, recently, has increased demand for natural bioactive ingredients, when incorporated into the packaging can present antioxidant and antimicrobial effects about packaged product [6], [8], [9], [10].

Among these substances, propolis has great potential to be added to the packaging, since its high antimicrobial and antioxidant activity has been confirmed in several studies [8], [9].

In this context, the aim of this study was to investigate the effect of incorporation of extract of red propolis (ERP) and licuri cellulose nanocrystals (CNCs) on the mechanical, barrier and bi-functional properties of the composites films based glycerol and cassava starch.

## Materials and Methods

### 2.1. Material

*Licuri* leaves used for extraction of CNCs were collected from farms (Cruz das Almas, Bahia, Brazil, located about 12 degrees south latitude and 43 degrees west longitude). The red propolis was donated by apiaries (Aracaju, Sergipe, Brazil). No specific permissions were required for these locations/activities. The studies did not involve endangered or protected species and provide. Cassava starch was donated by Cargill Agrícola S.A. (Porto Ferreira, Bahia, Brazil). Polyethylene was donated by Braskem S.A. (Camaçari, Bahia, Brazil). The cheese and butter were purchased at a local market (Salvador, Bahia, Brazil).

### 2.2. Licuri Cellulose Nanocrystals (CNC_S_) Preparation

Sulfuric acid hydrolysis of the leaves *licuri* was performed as described previously in the literature and in our previous works, with minor modifications. [13], [14], [15] Briefly, leaves *licuri* were ground and subjected to four washes using NaOH (80°C/4 hours). After bleached treatment of the cellulose, using NaOH and NaClO2 (sodium chlorite), the resulting material was ground until a fine particulate was obtained. Then, 10.0 g of cellulose was added to 160.0 mL of 64 wt% sulfuric acid under strong mechanical stirring. Hydrolysis was performed at 50°C for about 20 min. After hydrolysis, the dispersion was diluted twofold in water, and the suspension was washed using three repeated centrifuge cycles. The last washing was conducted using dialysis against deionized water until the dispersion reached a pH of 5–6. A stable suspension of CNCs was obtained through sonication for approximately 5 min.

### 2.3. Extract of the Propolis (ERP) Preparation

To prepare the extract, propolis was crushed and ground, adding grain alcohol 70%. The mixture was kept in a water bath temperature of 70°C under constant stirring for a period of 30 minutes. The extract was centrifuged at 4400 rpm, the temperature of 10°C for 10 minutes. The supernatant was filtered and then stored in amber vial at a temperature of 10°C.

### 2.4. Films Preparation

Sample films were prepared by casting a mixture of cassava starch (4 g) and glycerol (1 g) in a suspension containing 100 mL of distilled water. [3] The films were prepared by adding the aqueous dispersion of CNCs and ERP in the desired quantity. A series of cassava starch films plasticized with glycerol were prepared with CNC contents of 0–1.0 wt%, and ERP contents of 0.4–1.0 wt%, according to a central composite experimental design. The samples were heated to 70°C under constant stirring and with dehydration under renewable circulated air (35±5°C) in Petri plastic dishes. Samples were stored at 23°C and 75% relative humidity (RH) for 10 days prior to testing [6], [16].

### 2.5. Licuri Nanocellulose (CNCs) and Films Characterization

Transmission electron microscopy (TEM) was used to characterize the CNCs. TEM images were taken using a FEI Tecnai G2-Spirit with a 120-kV acceleration voltage. Diluted water suspensions of the nanowhiskers (0.01% wt/v) were deposited on a carbon–formvar-coated copper grid (300-mesh). The samples were subsequently stained with a 2% uranyl acetate solution. The length and width of the CNCs were obtained from several TEM images (50 images) [7].

Water activity (Aw) of the films was measured with a Decagon, Aqualab Lite. Pure water (Aw of 1.000±0.001%) and LiCl (Aw of 0.500±0.015%) were used as calibration standards. Preconditioned samples (4 cm^2^) were cut from the center of the films and evaluated in triplicate.

The moisture contents of films were determined by measuring their weight loss upon drying in an oven at 105°C until they reached a constant weight (dry sample weight). Samples were analyzed at least in triplicate, and results are expressed as percentage (g/100g) moisture contents of samples.

Mechanical properties of the films were obtained using an Emic Universal Testing Instrument (Model DL2000), operated as specified in the ASTM standard method D882-00. [17] Film strips of 8 cm×2.5 cm (length×width) were cut from each preconditioned sample and mounted between the grips of the machine. The initial grip separation and crosshead speed were set to 50 mm and 12.5 mm/min, respectively. At least 10 replicates of each specimen were averaged together. Values were analyzed of elastic modulus (*E*), tensile strength (σ) and elongation at break (ε) in films [17].

The water vapour permeability (WVP) of the films gravimetrically according to the methodology proposed by ASTM method E96-80 has been determined [18].

The determinations of total phenolic compounds (TPC) of the samples of the films were determined by spectrophotometry. The result was expressed as gallic acid equivalents (AG/g mg) calculated by curve constructed with varying acid concentrations 25–300 mg/mL (R2 = 0.99491) [19].

### 2.6. Antimicrobial Efficacy of the Films

To evaluate the antimicrobial activity of the films was used cheese rennet type. The cheese was sliced and submitted the incidence of UV light in a laminar flow chamber for 15 minutes, in order to reduce the initial microbial load. The samples were packed with active film containing highest concentration of both additives (F9-0.70% of ERP and 0.50% of CNCs), and stored in the refrigerator at a temperature of 7±2°C, along with the controls: samples packed with cassava starch films (without propolis; C1), with low-density polyethylene (LDPE; C2), and without any package (C3). The polyethylene film (0.089±0.011 mm) were extruded using a twin screw extruder. Samples were analyzed periodically for counting Staphylococci Coagulase positive (CSCP) for up to 28 days [20].

The formulation containing highest concentration of both additives (F9-0.70% of ERP and 0.50% of CNCs), was used to the evaluate the antimicrobial and antioxidant efficacy of films by presenting better mechanical characteristics and barrier.

### 2.7. Antioxidant Efficacy of the Films

The oxidative stability from a packaged product was carried out using peroxide value (PV) content at 0, 7, 15, 30, 45 and 60 days. Butter samples packed with cassava starch films (without propolis; C1), with low-density polyethylene (LDPE; C2), and without any package (C3) were used as controls. The PV was determined by titration according to Association of Analytical Communities (AOCS, 1997) methodology [6], [21].

### 2.8. Statistical Analysis

A central composite experimental design was used to evaluate the influence of concentration differences of the ERP and CNCs incorporated in bio-based films. ERP (%, w/w; X1) and CNCs (%, w/w; X2) were chosen as independent variables. The real and coded values of these variables are shown in Table 1. The Aw, TPC, WVP, *E*, σ and ε were used as dependent variables (Y).

**Table 1:** Values of the independents variables added to films as experimental design.

Film formulations	Coded values		Real values (%)	
	ERP (X_1_)	CNCs (X_2_)	ERP (X_1_)	CNCs (X_2_)
**F1**	–1.00	–1.00	0.50	0.15
**F2**	–1.00	+1.00	0.50	0.85
**F3**	+1.00	–1.00	0.90	0.15
**F4**	+1.00	+1.00	0.90	0.85
**F5**	–1.41	0.00	0.40	0.50
**F6**	+1.41	0.00	1.00	0.50
**F7**	0.00	–1.41	0.70	0.00
**F8**	0.00	+1.41	0.70	1.00
**F9** a	0.00	0.00	0.70	0.50
**F10** a	0.00	0.00	0.70	0.50
**F11** a	0.00	0.00	0.70	0.50

a *central points;* ERP = extract of red propolis; CNC_s_ **=** *licuri* cellulose nanocrystals.

A central composite experimental design with four axial points (R = 1.41) and three replications at the center points (for a total of 11 experiments) was employed (Table 1). The second-degree polynomials were calculated to estimate the response of the dependent variable (Stat, Inc., Minneapolis, MN) using eq 1:
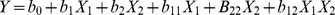
where Y is the predicted response, X1 and X2 are the independent variables, b0 is the offset term, b1 and b2 are the linear effects, b11 and b22 are the squared effects, and b12 is the interaction term. A film without ERP and CNCs (C 0) and the other without ERP (C 1) were used as controls for comparison of results.

The results of the evaluation of the antimicrobial efficacy of the antioxidant and films were analyzed by ANOVA using a statistical program StatSoft v.7 (StatSoft, Inc., Tulsa, Okla., USA). The Tukey test was used to evaluate average differences (at a 95% confidence interval).

## Results and Discussion

**Figure 1:**
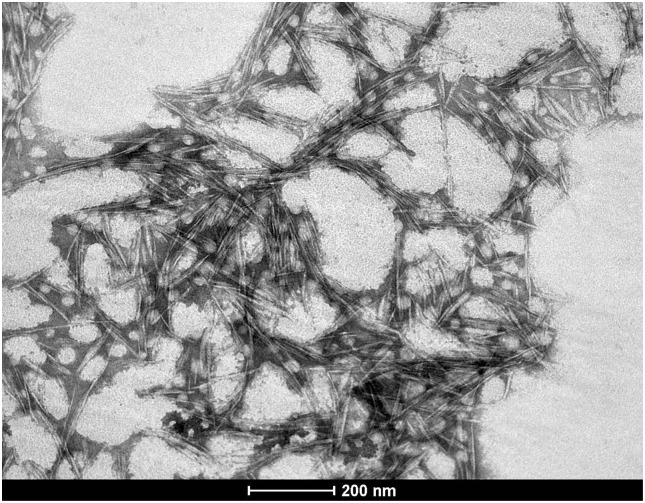
TEM image of licuri cellulose nanocrystals (CNC_s_).

Figure 1 is a TEM image of the cellulose nanocrystals from licuri leaves. Nanoparticles can be observed in the image and are represented by both aggregate and individual fibrils that are commonly found in TEM images of cellulose nanocrystals extracted from natural fibers. [11], [15] The *licuri* fiber used to extract the nanocellulose was composed of approximately 68% cellulose, 15.9% lignin and 8% hemicellulose.

The nanocellulose was generated in an aqueous suspension at a concentration of 0.00117 g/ml, with a yield of 33% relative to the mass of cellulose pulp used and 5.7% relative to the ground dry weight of the *licuri* leaves.

The isolated cellulose nanocrystals had a mean length of 157±24 nm and a mean diameter of 5.7±1.6 nm, resulting in an aspect ratio (L/D) of 27. Notably, there are no data in the literature on the nanocellulose extracted from licuri, but these values are in the range for nanocrystals from other lignocellulosic fibers that have great potential for use as reinforcement in biodegradable films. [10], [14] *Licuri* is a palm tree that originates in the semi-arid region of Brazil. This species is an abundant source of lignocellulosic fibers with high cellulose content and low cost, adding value to the supply chain, which is mainly familial.

When incorporated in different concentrations as additives in cassava starch and glycerol films, licuri cellulose nanocrystals (CNCs) and alcoholic aqueous extract of red propolis (ERP) significantly influenced the mechanical properties, barrier quality and simultaneous bi-functionality of the films.

**Figure 2:**
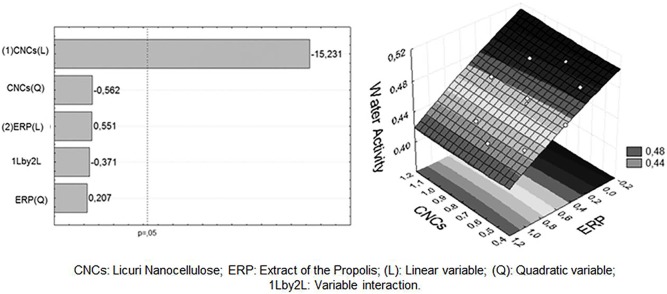
Pareto graph (A) and response surface (B) generated between CNCs, Water Activity and ERP.

**Figure 3:**
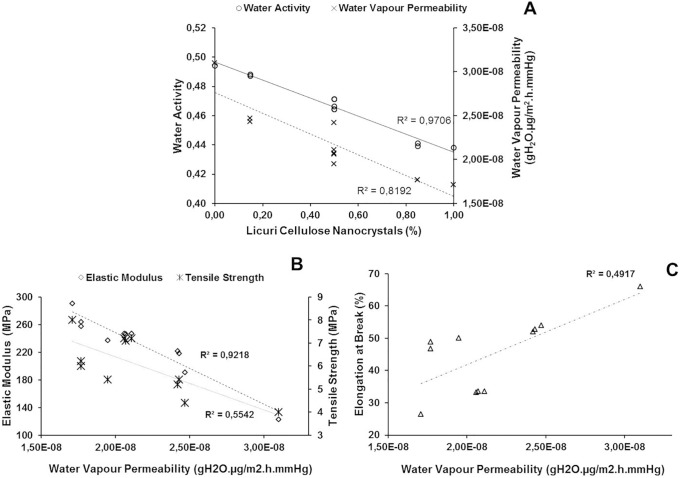
Linear correlation between the CNCs and the physico-chemical properties (A) and WVP and mechanical properties (B and C) of the films.

The Aw values of the various film formulations ranged from 0.438 to 0.494, depending on the concentration of incorporated CNCs and ERP additives (Table 2). The Pareto chart and response surface plot (Figure 2) indicate that the cellulose nanocrystals had a significant effect (p<0.05) on the water activity (Aw) of the films and show a linear inverse relationship between the increase in CNCs and decrease in the Aw (R2 = 0.9706, Figure 3A), as confirmed by the equation generated for the model (Equation 2).

**Table 2:** Properties of the films formulations with different contents of ERP and CNCs.

FilmFormulations	Aw	M (%)	TPC (mg AG/gof film)	WVP×10^−8^(gH_2_O.µg/m^2^.h.mmHg)	Mechanical properties		
					*E* (Mpa)	σ (Mpa)	ε (%)
**C0**	0.522±0.002	11,01±0,97	0.00	4.16±0.07	107.20±9.43	3.70±0.34	68.00±5.67
**C1**	0.462±0.002	10,61±0,67	0.00	1.86±0.06	296.20±14.86	7.80±0.45	22.80±5.22
**C2**	-	-	-	1.00–1.50	102.0–240.0	6.9–16.0	100.0–800.0
**F1**	0.487±0.004	10,23±1,09	68.42±1.09	2.43±0.02	217.80±32.51	5.40±0.89	52.80±6.10
**F2**	0.441±0.003	9,55±0,98	64.53±0.41	1.77±0.04	257.20±44.06	6.20±0.84	46.80±5.02
**F3**	0.488±0.004	10,08±0,73	86.32±0.48	2.47±0.01	190.80±42.76	4.40±0.55	54.00±7.35
**F4**	0.439±0.006	10,43±1,49	83.68±0.39	1.77±0.03	264.00±38.12	6.00±1.41	48.80±8.90
**F5**	0.466±0.003	11,46±0,91	61.28±0.56	2.42±0.03	221.40±19.51	5.20±0.84	52.00±8.60
**F6**	0.471±0.009	10,75±0,67	98.33±0.44	1.95±0.03	237.00±34.66	5.40±0.55	50.00±7.35
**F7**	0.494±0.004	10,09±1,07	83.24±0.97	3.10±0.07	122.40±8.73	4.00±0.71	66.00±6.93
**F8**	0.438±0.002	9,38±0,55	79.44±0.71	1.71±0.05	290.20±58.52	8.00±0.71	26.40±6.54
**F9** a	0.464±0.001	11,27±0,76	81.15±0.78	2.06±0.03	246.60±32.44	7.20±0.84	33.20±7.69
**F10** a	0.464±0.001	11,44±1,53	82.26±0.15	2.11±0.01	247.00±34.73	7.20±1.64	33.60±8.05
**F11** a	0.471±0.004	11,60±1,38	82.18±0.59	2.07±0.03	246.20±15.35	7.20±1.30	33.60±9.21

a *central points;* C0 = cassava starch films without propolis and cellulose nanocrystals***;*** C1 = cassava starch films without propolis; C2 = polyethylene film [35]; Aw = water activity; M = moisture; TPC = total phenolic compounds; WVP = water vapor permeability; *E* = elastic modulus; σ = tensile strength; ε = elongation at break; ERP = extract of the red propolis; CNCs = *licuri* cellulose nanocrystals.



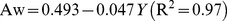
(2)Statistical analyses indicated that the addition of ERP did not significantly interfere (p>0.05) with the Aw values (Figure 2).

There was an 11% reduction in the Aw values of the films with 0.5% CNCs (C1 - cassava starch films without propolis) compared to the control films (C0 - cassava starch films without propolis and cellulose nanocrystals), indicating that CNCs reduce water availability within the matrix of the nanocomposite material and have a relevant role in controlling water availability due to the increase in the number of hydrogen bonds with the polymeric matrix and plasticizer [7].

Considering that water migrates from areas of high Aw to areas of low Aw, it is essential that films for food-product packaging have low values for this parameter because such films can reduce the amount of water available for the growth of microorganisms or unwanted chemical reactions during product storage.

The interference of the cellulose nanocrystals was also observed in the moisture and water vapor-permeability (WVP) results (Table 2). The formulations that had the highest concentrations of CNCs also had the lowest WVP rates and moisture. There was a 55% reduction in the WVP for the films with 0.5% CNCs (C1) compared to the control film (C0). Regarding moisture reduction was 4%.

The moisture is an important parameter to measure, is the basis for several characteristics of the films, including the mechanical properties and water vapour permeability. The moisture of the films ranged 61.28–98.33%, with differences between the formulations, however, there was a very low correlation coefficient of this parameter with the water vapour permeability (r^2^ = 0.007), elongation (r^2^ = 0.40) and tensile strength (r^2^ = 0.0005), indicating that this parameter has little influence on the properties of the films analyzed.

The WVP values and the CNC concentrations of the films showed an inverse proportional and linear relationship, similar to the Aw values, with R^2^ = 0.9706 (Figure 3A). At any concentration, the cellulose nanocrystals in the films are intended to reduce the WVP by providing a physical barrier, reducing the free spaces in the polymer matrix, restricting the passage of vapors and inhibiting water absorption. The degree of crystallinity of the cellulose nanoparticles and the strong hydrogen bonds formed within the matrix restrict access and hence the permeability of water through the film [14], [15], [22].

The correlation between the concentration of the ERP of the films and the WVP was not significant (p>0.05), although other studies [23], [24] have shown a reduction in the WVP of polymer matrices with the addition of natural products rich in phenolic compounds. These compounds can form hydrogen bonds with the reactive groups in the polymer matrix, lowering the rate of water permeation by limiting the availability of hydrogen groups to form hydrophilic bonds with water [23].

Comparing the obtained films with polyethylene films that are commonly utilized, it is noted that biodegradable films showed higher elastic modulus and tensile strength greater than the polyethylene film proving to the mechanical reinforcement by the addition of nanocrystals (Table 2).

**Figure 4:**
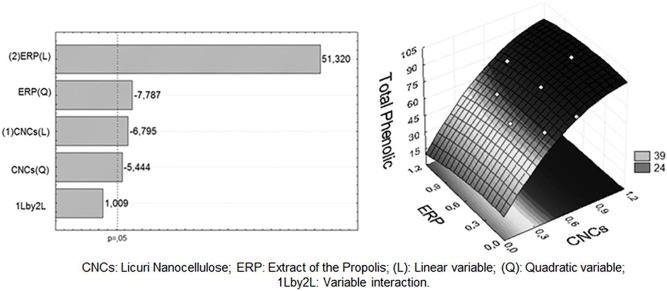
Pareto graph (A) and response surface (B) generated between CNCs, Total Phenolic and ERP.



With respect to total phenolic compounds (TPC) derived from the addition of ERP to the films, the Pareto chart and response surface (Figure 4) show that the concentrations of ERP and CNCs significantly influence (p<0.05) this independent variable. As expected, as the concentration of ERP in the films increases, there is an almost linear and directly proportional increase in the TPC, indicating that there is a good incorporation of the propolis and its active compounds in the nanocomposite. The equation for this model (Equation 3) indicates that the TPC content in the films does not depend on the interaction of the independent variables studied but is only influenced by the variables in an isolated manner.

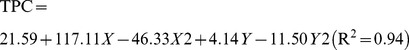
(3)The presence of CNCs also played an important role in the mechanical properties of the films. The values obtained for the mechanical properties of the films did not fit well with the adopted mathematical model; however, the results indicate that the elastic modulus and tensile strength values for the films increased 176% and 111%, respectively, with the use of 0.5% CNC (C1). These results demonstrate that cellulose nanocrystals can be used to reinforce biodegradable matrices, even at low concentrations.

The tensile strength values can be defined as the maximum resistance provided by the films when subjected to tension, and larger values may be related to the interaction between the polymer chains and the cellulose nanocrystals, forming a structure that is more resistant to tension [15].

The addition of ERP alone did not affect the mechanical properties of the films: similar results were obtained for the F 7 (without ERP) and control films (C 0) (Table 2). However, the results for all the formulations indicated that the addition of ERP combined with CNCs, even at low concentrations, has an opposite effect than the CNCs alone. In the formulations with the same CNC content and different concentrations of ERP (F1 and F3), increases in the proportion of ERP further reduce the elastic modulus and tensile strength values of the films.

A reduction in the strength of films with an increase in the concentration of oils and extracts from natural products has been reported in other studies [25], [26] and is related to the replacement of polymer components by natural components, resulting in a less-resistant matrix [25] Phenolic derivatives derived from propolis extracts result in weakened interactions between the other components of the nanocomposite.

The CNCs also had an effect on the elongation at break of the films. With the addition of 0.5% CNCs to the films, there was a 66% reduction in the deformation of the films (C1). The addition of nanocrystals favors the formation of a more compact structure, which increases stiffness and reduces the flexibility of the films. [7] This behavior has also been reported in other studies and can be used as an indication of good interactions between the different film components, [22] forming a continuous network of nanocrystals, connected via strong hydrogen bonds [27].

Figure 3B and 3C shows the linear correlation between the WVP values and mechanical properties of the films. There is a linear and inverse proportional correlation between the WVP and elastic modulus of the films (R^2^ = 0.9218). The increased permeability of the films significantly reduced the film’s stiffness and hence its ability to stretch, i.e., its modulus of elasticity. This correlation is extremely important because it indicates that the addition of CNCs can linearly reduce the WVP and increase the elastic modulus of the films, making the film more competitive with standard products. The film (F9) containing intermediate levels of ERP (0.70%) and CNCs (0.50%) was selected for food packaging and to evaluate the bi-functional efficacy of the films because it had the combination of values for Aw, WVP, TPC and mechanical properties that together provided the most satisfactory film characteristics.

### 3.1. Antimicrobial Efficacy of the Films

**Figure 5:**
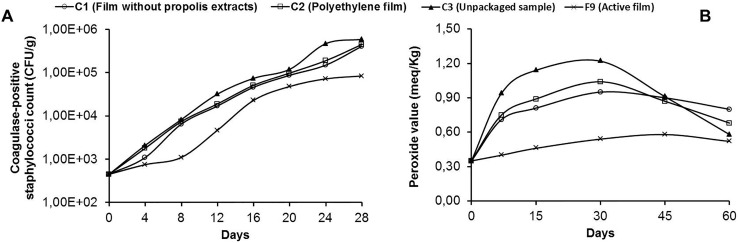
Antimicrobial efficacy (A) and antioxidant (B) the active film during products storage.

Figure 5A shows the number of CFUs of coagulase-positive staphylococci in cheese packed using films C1, C2, C3 and F9 during 28 days of storage. The active film F9 containing 0.70% and 0.50% of ERP and CNCs, respectively, showed an approximately 1 log reduction in the number of these microorganisms at 4, 12, 20 and 28 days compared to all the controls. As expected, the exposed cheese (3 C) showed the highest number of CFUs at all the periods analyzed. There were no significant differences (p>0.05) between the number of CFUs in the cheese packed in the polyethylene film (C 2) and the starch film without propolis (C 1), demonstrating that these films play equivalent roles in protecting the product from contamination by coagulase-positive staphylococci (Figure 5A, Table 3). This result reflects the addition of nanocellulose to the biodegradable film, which reduced the Aw and WVP of the film, which are extremely important properties in controlling microbial growth in foods.

**Table 3:** Effect Antimicrobial and antioxidant of active film during products storage.

Film formulations	Count of coagulase-positive staphylococci (CFUs) in cheese, during 28 days of storage (days)							
	0	4	8	12	16	20	24	28
**C1**	4,5E+02^(a)^	1,1E+03^(a)^	6,5E+03^(b)^	1,7E+04^(b)^	4,6E+04^(b)^	8,7E+04^(b)^	1,5E+05^(b)^	4,1E+05^(b)^
**C2**	4,5E+02^(a)^	1,8E+03^(a)^	7,4E+03^(b)^	1,9E+04^(b)^	5,1E+04^(b)^	9,8E+04^(b)^	1,9E+05^(b)^	4,5E+05^(b)^
**C3**	4,5E+02^(a)^	2,1E+03^(a)^	8,3E+03^(a)^	3,2E+04^(a)^	7,4E+04^(a)^	1,2E+05^(a)^	4,7E+05^(a)^	5,9E+05^(a)^
**F9**	4,5E+02^(a)^	7,5E+02^(b)^	1,1E+03^(c)^	4,7E+03^(c)^	2,3E+04^(c)^	4,8E+04^(c)^	7,2E+04^(c)^	8,4E+04^(c)^
**Film formulations**	**Peroxide index (PI) of the butter (meq/Kg) during storage (days)**							
	**0**	**7**	**15**	**30**	**45**	**60**	**-**	**-**
**C1**	0.35^a^	0.71^a^	0.81^b^	0.95^b^	0.90^a^	0.80^a^	-	-
**C2**	0.35^a^	0.75^a^	0.89^a,b^	1.04^a,b^	0.87^a^	0.68^a,b^	-	-
**C3**	0.35^a^	0.94^a^	1.14^a^	1.22^a^	0.91^a^	0.58^a,b^	-	-
**F9**	0.35^a^	0.40^b^	0.46^c^	0.54^c^	0.58^b^	0.52^b^	-	-

Means with the same letters in the same columns presented no statistical difference (p>0.05) according to Tukey’s test.

C1 = film without propolis; C2 = film of low-density polyethylene; C3 = product without any package; F9 = active film (starch, glycerol and 0.5% licuri cellulose nanocrystals).

Despite the increase in the number of CFUs of coagulase-positive staphylococci during the shelf life of the packaged product, active films played an active role in inhibiting the growth of these microorganisms, given that the number of CFUs was low over the analysis period, indicating that the film has an antimicrobial effect on the microorganisms in the matrix studied. The high initial load of staphylococci in cheese (4.5E+02 CFU/g) can be reduced because of the antimicrobial efficacy of the film. Moreover, the intrinsic factors (fat, protein, water, pH and preservatives) and extrinsic factors (temperature, vacuum packaging and microbial characteristics) of the food can also influence the sensitivity of the bacteria and reduce the effectiveness of the films [28].

The number of CFUs indicates that the ERP in the active film had inhibitory activity against the microorganism evaluated, which reached its lowest number of CFUs over the entire storage period.

The antimicrobial activity of alcoholic extracts of propolis on Gram-positive bacteria has been reported in several studies [24], [25], [29] and can be attributed to the presence of phenolic compounds that inhibit bacterial growth via inhibition of the bacterial RNA polymerase and disruption of the bacterial cell membrane and cytoplasm. [8], [9], [30] In products with a low initial microbial load, the antimicrobial effect of the active films is greater and more easily observed because for these products, the packaging should only provide an inhibitory effect by slowing bacterial growth, whereas in products with high microbial loads, active films must have both an inhibitory and bactericidal effect to eliminate pre-existing microorganisms.

The action of antimicrobial films can occur via the diffusion of active compounds into the food or may only be the result of direct contact between the food and the surface of the film, without diffusion of active compounds. [31] Importantly, the interaction between polymer groups and the active compounds of the embedded agent can reduce or even prevent the diffusion of active compounds into the system, reducing the effectiveness of the compounds. Thus, it is evident that the inhibitory effect of the bioactive compounds also appears to be associated with the macromolecules used in the films’ formulation, and it is therefore essential to understand the macromolecule’s properties and how they interact with the active compounds [32].

Similar results were found in a study that evaluated the antimicrobial activity of starch films activated with oregano oil in storage of fresh beef. The authors observed that films with addition of 1.5% of oregano oil fell 2 log reduction counts of mesophilic and pseudomonas [33].

### 3.2. Antioxidant Efficacy of the Films

The antioxidant efficacy of the film containing 0.70% ERP and 0.50% CNCs (F 9) was evaluated for butter storage using the nanocomposite material. The active film with propolis significantly slowed the oxidation process of butter (p<0.05) compared to controls over the 60-day storage period (Figure 5B, Table 3). The results in this investigation are in agreement with studies of Souza et al. [6] who reported that the PV of palm oil packaged with starch film containing mango and acerola pulp added as antioxidants were lower than those of the control during storage. At 60 days of storage, the PV of the butter packaged in the active film (F 9) increased 49%. The PV of the butter packaged in the film without propolis extracts (C 1) and in low-density polyethylene (C 2) increased 129% and 94%, respectively, after 30 days of storage. At this time, the PV of the butter packaged in the F 9 film was approximately 3 times lower, showing a large reduction in the induction period for oxidation.

The PV of the samples packed in the control films C 1 and C 2 were not significantly different (p>0.05) from the unpackaged sample (C 3) at 7, 45 and 60 days. These results suggest that these films did not provide any protection to the product during storage (Figure 5B).

Notably, in the first 30 days of storage, the butter wrapped in the control film (C 2 - starch, glycerol and nanocellulose) had a lower PV than the sample stored in a polyethylene film (C 1), indicating that the nanocomposite film without propolis extract was more effective in controlling oxidation of the product than the synthetic film. These results suggest that the mechanical reinforcement achieved by the addition of nanocellulose to the film reduces both the film’s permeability to water vapor and also its permeability to gases, which would have a direct effect on the oxidation of the stored product. These results are in agreement with some studies that have shown that oxygen can permeate the film and react preferentially with compounds present in the film formulation, allowing the packaged product to be preserved for a longer period of time. For films containing yerba mate extract and mango pulp used to pack palm oil, the authors reported a significant maximum reduction of 25,40% (between formulations) and 87,40% (between formulations and control) (p<0.05) for the oxidation of oil after 45 days of storage [34].

The main purpose of using antioxidants in packaging for food lipids is to delay a significant accumulation of free radicals and thus improve oxidative stability. The results of this study suggest that the protection of packaged products against oxidation can be attributed to the concentration-dependent radical scavenging activity of antioxidant compounds present in film-forming dispersions (Table 3). This outcome occurred because antioxidants act by inhibiting or interrupting the mechanism of lipid auto-oxidation of free radicals. The protective effect against lipid oxidation is likely due to a physical and synergistic process mostly from the total polyphenols and total flavonoids presents in propolis.

## Conclusions

The results indicate that biodegradable films derived from starch and glycerol with the addition of cellulose nanocrystals extracted from licuri and alcoholic extracts of red propolis can be a competitive alternative in reducing the use of synthetic packaging films in the storage of some foods. These films may have a simultaneous antimicrobial and antioxidant bi-functional effect. The highly ordered structure of cellulose nanocrystals increases the mechanical strength of the films and reduces the moisture, water activity and water-vapor permeability, which are both critical properties in food packaging. The active film containing 0.70% and 0.50% of the additives ERP and CNCs, respectively, limited the growth of coagulase-positive staphylococci in cheese and slowed the oxidation of butter. Additional studies are therefore needed to evaluate the effect of these films on other food matrices, in which the storage conditions and the characteristics of products and their possible interactions with the films should demonstrate the true effectiveness of the active packaging. The potential of bi-functional films for use in medical and clinical areas and for cosmetics and pharmaceutical products should also be evaluated, increasing the potential applications for these nanocomposite materials.
